# Possible Beneficial Effects of Fresh Pomegranate Juice in SARS-CoV-2 Infection Conditions

**DOI:** 10.1155/2022/5134560

**Published:** 2022-03-05

**Authors:** Saleem Ali Banihani

**Affiliations:** Department of Medical Laboratory Sciences, Jordan University of Science and Technology, Irbid 22110, Jordan

## Abstract

Rather than the prophylactic vaccination, any effective synthetic, natural, or nutritional therapy or regimen that may cure or remedy, albeit partially, the complications of SARS-CoV-2 should be highly acknowledged. Here, we reviewed and discussed possible beneficial biological effects of pomegranate juice in such diseased condition of viral infection based on the current published evidence (direct and indirect) and owing to the robust evidence that fresh pomegranate juice is highly rich with unique bioactive compounds that are approved in various occasions to be effective in several chronic diseased conditions. All related references that serve our aim are accessed through available electronic databases, particularly PubMed and Scopus. In summary, there is accepted evidence that pomegranate juice may be beneficial in SARS-CoV-2 infection conditions, especially for patients with the clinical history of chronic diseases such as hypertension, cardiovascular disease, diabetes, and cancer. However, the interventional studies that directly probe and confirm the effectiveness of fresh pomegranate juice in the management of SARS-CoV-2 infection are mandatory.

## 1. Introduction

Since December 2019, the acute respiratory syndrome coronavirus 2 (SARS-CoV-2) cases reached more than 250 million with more than 5 million deaths worldwide [1]. Still, this number is increasing every day [1]. Other than the used prophylactic vaccines, to date, the potential therapy to manage SARS-CoV-2 infection conditions is still an ongoing global challenge with a very limited specific pharmacological therapy which has been identified to be potentially effective in such diseased circumstances, especially with the evident vaccine breakthrough infections with different variants of SARS-CoV-2, making this pandemic the worst for this generation since the Spanish flu of 1918 [2].

Therefore, any approved natural product or even food regimen that may cure or reduce, albeit partially, the complications of SARS-CoV-2 infection should be highly acknowledged. One such possible example that could be helpful in the management of this unique diseased situations is fresh pomegranate juice.

In actual fact, our attention to review fresh pomegranate juice in ameliorating SARS-CoV-2 infection conditions was not random, but it is due to several published reports that correlate the severity and mortality rate of coronavirus disease 19 (COVID-19) with the clinical history of the infected patients [3–6]. It has been shown that patients with COVID-19 and with the clinical history of chronic diseases/conditions such as coronary heart disease [7], hypertension [8], cancer [9, 10], and diabetes were found to develop more serious and critical cases, and hence markedly increase rates of death among these people [6, 11, 12]. In addition, in general, a number of research studies have revealed a valuable benefit of pomegranate juice in management of viral infection, including influenza [13–15].

In effect, the potential health benefits of pomegranate juice is due to its antihypertensive [16], antiatherogenic [17], anti-inflammatory [18, 19], anticancer [20], and antidiabetic effects [21]. Such imperative biological activities of pomegranate juice are due to its unique chemical composition, which includes a wide range and powerful dietary phytochemicals such as flavonoids (e.g., quercetin, phloridzin, and catechin) [22, 23] and polyphenols (e.g., anthocyanins, tannins, gallic acid, ferulic acid, coumaric acids, and chlorogenic acid) [24]. Also, here, it is worth mentioning that these substantial phytochemicals have let pomegranate juice to exhibit a very strong antioxidant activity that is greater than the renowned antioxidants, for example, from red wine or green tea [25].

## 2. Pomegranate Juice against Viral Infection

Studies have revealed the effectiveness of pomegranate juice or its bioactive compounds against different types of viruses, for example, pomegranate juice was found to inactivate human immunodeficiency virus (HIV) may be by blocking the HIV virus binding to the cluster of 4 (CD4), which is a glycoprotein present on the surface of several types of white blood cells such as T helper cells and monocytes [13]. The same study suggested that at least one of the bioactive compounds in pomegranate juice bound tightly, or irreversibly, to the CD4 binding-site on envelope glycoprotein GP120 [13]. In addition, an experimental study showed that punicalagin, a polyphenol in pomegranate juice, has been identified to have a potential virucidal activity against herpes simplex virus in epithelial Vero host cells [26]. Moreover, another experimental study on the effect of pomegranate juice on the infectivity of foodborne viral surrogates showed that 20 minutes incubation with pomegranate juice powder at approximately 4 or 8 mg·mL^−1^ resulted in titer reductions of 0.79, 3.12, and 0.23 log (10) PFU mL^−1^ for murine norovirus 1, feline calicivirus (strain F9), and MS2 bacteriophage, respectively [27].

In particular, pomegranate juice has revealed a potential antiinfluenza activity, for example, Sundararajan and coworkers (2010) found that five minutes treatment with pomegranate juice powder extract at 800 *μ*g·mL^−1^ significantly reduced (by approximately 3 log) the titers of influenza viruses: influenza A virus subtype H1N1, PR8 virus, influenza A virus subtype, and reassortant H5N1 avian influenza virus derived from a human isolate [14]. In the same study, Sundararajan and coworkers have demonstrated that the antiinfluenza activity of pomegranate juice is significantly modulated by some alterations in envelope glycoproteins [14]. Moreover, an experimental in vitro study conducted by Madjid and coworkers revealed that pomegranate polyphenol extract from pomegranate juice significantly inhibited the replication of influenza A virus subtype H3N2 (human influenza A/Hong Kong) [15]. The same researchers evaluated some polyphenols (luteolin, ellagic acid, punicalagin, and caffeic acid) in pomegranate polyphenol extract and found that punicalagin is the main bioactive antiinfluenza compound. It has been found to block the replication of virus RNA and inhibit the agglutination of chicken red blood cells by the virus [15]. This evidence is strongly revealing the effectiveness of pomegranate juice in viral infection conditions, particularly in influenza.

Newly published studies have revealed that some anthocyanins and tannins rich plants such as pomegranate could be very beneficial to cure COVID-19 [28, 29]. In particular, structurally, it was found that some polyacylated anthocyanins (e.g., phacelianin, cyanodelphin, gentiodelphin, and tecophilin) and hydrolysable tannins (e.g., tercatain, pedunculagin, and castalin) may inhibit SARS-CoV-2 papain-like protease and hence the replication of virus [28, 29].

## 3. Possible Beneficial Effects of Pomegranate Juice in COVID-19 Patients with Chronic Diseases

### 3.1. Pomegranate Juice and COVID-19 Patients with Hypertension

The activity of serum angiotensin-converting enzyme (ACE) reduced by approximately 36% in seven out of ten hypertensive patients after two weeks following pomegranate juice consumption at 50 mL per day [30]. Also, the similar in vitro inhibitory effect (31%) of pomegranate juice on activity of serum ACE was observed [30]. As a consequence of such enzymatic inhibition, there was a reduction in systolic blood pressure by approximately 5% in the recruited patients [30]. A randomized placebo-controlled study conducted on fifty-one healthy adults revealed that consumption of 330 mL per day pomegranate juice, for 4 weeks, significantly reduces both systolic and diastolic blood pressures [31]. Another randomized clinical trial has shown that hemodialysis patients (*n* = 101) received 100 mL of pomegranate juice three times a week, for 1-year, and had significant improvement in their pulse pressure and systolic blood pressure compared to the placebo group [32]. Moreover, a later randomized controlled trial has shown that patients administered natural pomegranate juice at 500 mL per day, for one week, had lower systolic and diastolic blood pressures [33]. Collectively, this evidence confirms the ACE inhibition activity and consequently the hypotensive response of pomegranate juice in the human body. Alternatively, in our recent published clinical trial, we have shown that the observed antihypertensive effect of fresh pomegranate juice in humans may be modulated by a reduction in the cortisol level [34].

Mechanistically, angiotensin-converting enzyme 2 (ACE2), a membrane-bound and a zinc-containing metalloenzyme, has been found to be present in several bodily organs such as the lung, arteries, small intestine, and kidneys [35]. In particular, at most, ACE2 is attached to the type II pneumonocytes in the alveoli, arterial smooth muscle cells, venous endothelial cells, and small intestinal absorptive cells (or enterocytes of the small intestine) [36]. Biochemically, ACE2 is an aminopeptidase that cleaves angiotensin II (AT-II), which is a vasoconstrictor, into angiotensin-(1–7), which is a vasodilator [35]. Very recently, ACE2 has been recognized as a receptor and an entry point for the acute respiratory syndrome coronavirus 2 into the host cells; therefore, it has a critical role in the onset of coronavirus disease 2019 [37].

Even though administration of ACE inhibitors was found to increase cardiac mRNA levels of ACE2, it has no effect on the activity of ACE2 in the experimental animal models [35, 38, 39]. Li et al. showed that treatment with captopril (brand name: Capoten), which is a commonly used ACE inhibitor, increases expression of ACE2 in rats with acute lung damage [40]. Also, it was documented that, in rats with acute respiratory distress syndrome, expression of AT-II and ACE activities increased, while activity of ACE2 and level of angiotensin-(1–7) decreased [41]. In fact, decreasing the amount of ACE2 will shift the balance of the renin–angiotensin–aldosterone system (RAAS) to promote the ACE–AT-II–AT-II receptor type I axis, leading to progression of inflammatory storms and lung injury [42]. Increased level of ACE2 will move the balance to the AT-(1–7)-Mas receptor axis, which has antioxidant as well as anti-inflammatory effects, and hence a cardiopulmonary protective consequence [42]. Accordingly, the above evidence suggested that even though ACE2 is considered as an entry point for SARS-CoV-2, increased expression of ACE2 by the effect of ACE inhibitors is suggested not to worse SARS-CoV-2 infection or COVID-19. In fact, alternatively, certain RAAS inhibitors may behave differently, for example, diabetic nephropathy models, chronic administration of aliskiren, a direct renin inhibitor, was found to be associated with a decrease in the expression of ACE2, which is suggested to be an interesting research route in context of infection by SARS-CoV-2 [43].

### 3.2. Pomegranate Juice and COVID-19 Patients with Diabetes

In our randomized clinical trials published in 2014 and 2020, we found that pomegranate juice administered at 1.5 mL per kg of bodyweight significantly reduces fasting serum glucose in patients with type 2 diabetes and people with impaired fasting glucose compared to control [21, 44]. Previous preclinical in vivo study has shown that pomegranate seed extract at approximately 0.45 g·kg^−1^ reduced fasting (12 hours fasting) blood glucose in diabetic models [45]. Also, punicic acid, an organic acid in pomegranate juice, at 1000 mg per 100 g of the fed diet, for 30 days, reduced fasting blood glucose in obese mice models [24, 46]. Moreover, other organic acids in pomegranate juice (ellagic acid, gallic acid, ursolic acid, oleanolic acid, and uallic acids) were identified to have antihyperglycemic response [47–50].

Mechanistically, pomegranate juice may exert its antihyperglycemic response via enhancing *β*-cell function, reducing insulin resistance, decreasing cortisol level [34], and minimizing oxidative stress state [24], which is an imbalance between oxidants (e.g., reactive oxygen species) and antioxidants [51, 52]. The later mechanistic route may be attributable to enhancing activity of vital antioxidant enzymes such as catalase, superoxide dismutase, and glutathione reductase [53], direct neutralization of free radicals such as hydroxyl radical or superoxide ion, decreasing synthesis of resistin [54], which is a hormone secreted from adipose tissue and found to be associated with type 2 diabetes [55], increasing metal binding activity [56], and activating or inhibiting transcriptional factors such as peroxisome proliferator-activated receptor gamma (PPAR-*γ*) and/or nuclear factor-*κ*B (NF-*κ*B) [24, 57, 58]. These results provide robust evidence to the beneficial effects of pomegranate juice in diabetic conditions, which therefore may provide beneficial effects to patients with diabetes and infected with SARS-CoV-2.

### 3.3. Pomegranate Juice and COVID-19 Patients with Cardiovascular Disease

Pomegranate juice has been presently known as a cardioprotective natural product due to its ability to reduce ACE activity [30, 59], thereby decreasing systolic and diastolic blood pressures (as explained above) [60], increasing total serum antioxidant capacity [61, 62], enhancing activity of certain antioxidant enzymes [63, 64], decreasing levels of plasma lipids [65, 66], reducing lipid peroxidation [65], decreasing H_2_O_2_-induced toxicity [67], and decreasing the uptake of oxidized low-density lipoprotein by macrophages [68, 69]. In addition, it was found to decrease thickness of intima media by macrophages and reduce the atherosclerotic lesion areas [70]. Moreover, pomegranate juice was found to enhance the biological action of nitric oxide and decrease inflammation [18, 19]. Therefore, it causes overall beneficial effects against the development of atherosclerosis and the consequent progression of coronary artery disease. Such cardioprotective effect of pomegranate juice could be valuable for patients with COVID-19 and with the clinical history of cardiovascular diseases.

### 3.4. Pomegranate Juice and COVID-19 Patients with Cancer

To date, there are more than 88 research studies and reports in the PubMed database that are linking pomegranate to cancer. Such research accumulation is attributable to the encouraging anticancer properties of pomegranate juice and its derived compounds [71]. In fact, pomegranate juice has been suggested to be a promising chemoprotective and/or chemotherapeutic agent against various types of cancer, mainly prostate [72–75], skin [76, 77], colon [78–80], breast [81–84], and lung cancer [20]. Mechanistically, in context of cancer, pomegranate juice has been identified as having antioxidant [73, 85, 86], anti-inflammatory [18, 80], antiproliferative [67, 87], and antitumorigenic [88, 89] effects, modulating different signaling pathways.

In effect, the anticancer biological properties of pomegranate juice may due to the presence of bioactive chemical compounds such as ellagitannins [90, 91], anthocyanins [28, 76, 92], hydrolysable tannins [92, 93], ellagic acid [75, 85, 94], luteolin [94], and punicic acid [82, 87, 95]. Accordingly, the anticancer properties of pomegranate juice and its derived compounds suggest a possible accommodating effect for cancer patients infected with SARS-CoV-2.

## 4. Conclusion

Collectively, in view of that pomegranate juice is an edible and safe natural product, there is accepted, however, indirect evidence that ingesting fresh pomegranate juice could be beneficial in SARS-CoV-2 infection conditions, especially in the conditions that coincide with the existence of the history of other chronic diseases such as hypertension, cardiovascular disease, diabetes, or cancer.

The current possible beneficial effect in pomegranate juice in viral infection conditions, including SARS-CoV-2 infection, may be due to the presence of several potential bioactive compounds (e.g., polyacylated anthocyanins and hydrolysable tannins). However, human interventional studies that recruit, for example, patients with SARS-CoV-2 and directly probe the effect of fresh pomegranate juice shall provide the direct and the robust evidence toward such implied effectiveness of pomegranate juice in such exceptional diseased condition.

## References

[1] WHO (2020). *WHO Coronavirus Disease (Covid-19) Dashboard*.

[2] Sanders J. M., Monogue M. L., Jodlowski T. Z., Cutrell J. B. (2020). Pharmacologic treatments for coronavirus disease 2019 (COVID-19). *JAMA*.

[3] Zheng Y.-Y., Ma Y.-T., Zhang J.-Y., Xie X. (2020). Covid-19 and the cardiovascular system. *Nature Reviews Cardiology*.

[4] Wang C. Z., Hu S. L., Wang L., Li M., Li H. T. (2020). Early risk factors of the exacerbation of coronavirus disease 2019 pneumonia. *Journal of Medical Virology*.

[5] Jordan R. E., Adab P., Cheng K. K. (2020). Covid-19: risk factors for severe disease and death. *BMJ*.

[6] Fang L., Karakiulakis G., Roth M. (2020). Are patients with hypertension and diabetes mellitus at increased risk for covid-19 infection?. *The Lancet Respiratory Medicine*.

[7] Ji H.-L., Zhao R., Matalon S., Matthay M. A. (2020). Elevated plasmin(ogen) as a common risk factor for covid-19 susceptibility. *Physiological Reviews*.

[8] Lippi G., Wong J., Henry B. M. (2020). Hypertension in patients with coronavirus disease 2019 (covid-19): a pooled analysis. *Polish Archives of Internal Medicine*.

[9] Xia Y., Jin R., Zhao J., Li W., Shen H. (2020). Risk of covid-19 for patients with cancer. *The Lancet Oncology*.

[10] Zhang L., Zhu F., Xie L. (2020). Clinical characteristics of covid-19-infected cancer patients: a retrospective case study in three hospitals within wuhan, China. *Annals of Oncology*.

[11] Zhou F., Yu T., Du R. (2020). Clinical course and risk factors for mortality of adult inpatients with covid-19 in wuhan, China: a retrospective cohort study. *The Lancet*.

[12] Guo W., Li M., Dong Y. (2020). Diabetes is a risk factor for the progression and prognosis of COVID‐19. *Diabetes*.

[13] Neurath A. R., Strick N., Li Y.-Y., Debnath A. K. (2004). Punica granatum(Pomegranate) juice provides an HIV-1 entry inhibitor and candidate topical microbicide. *BMC Infectious Diseases*.

[14] Sundararajan A., Ganapathy R., Huan L. (2010). Influenza virus variation in susceptibility to inactivation by pomegranate polyphenols is determined by envelope glycoproteins. *Antiviral Research*.

[15] Haidari M., Ali M., Ward Casscells S., Madjid M. (2009). Pomegranate (punica granatum) purified polyphenol extract inhibits influenza virus and has a synergistic effect with oseltamivir. *Phytomedicine*.

[16] Sahebkar A., Ferri C., Giorgini P., Bo S., Nachtigal P., Grassi D. (2017). Effects of pomegranate juice on blood pressure: a systematic review and meta-analysis of randomized controlled trials. *Pharmacological Research*.

[17] Rosenblat M., Volkova N., Borochov-Neori H., Judeinstein S., Aviram M. (2015). Anti-atherogenic properties of date vs. Pomegranate polyphenols: the benefits of the combination. *Food & Function*.

[18] Danesi F., Ferguson L. R. (2017). Could pomegranate juice help in the control of inflammatory diseases?. *Nutrients*.

[19] Ginsberg Y., Khatib N., Saadi N., Ross M. G., Weiner Z., Beloosesky R. (2018). Maternal pomegranate juice attenuates maternal inflammation-induced fetal brain injury by inhibition of apoptosis, neuronal nitric oxide synthase, and NF-*κ*B in a rat model. *American Journal of Obstetrics and Gynecology*.

[20] Husari A., Hashem Y., Zaatari G., El Sabban M. (2017). Pomegranate juice prevents the formation of lung nodules secondary to chronic cigarette smoke exposure in an animal model. *Oxidative Medicine and Cellular Longevity*.

[21] Banihani S. A., Makahleh S. M., El-Akawi Z. (2014). Fresh pomegranate juice ameliorates insulin resistance, enhances *β*-cell function, and decreases fasting serum glucose in type 2 diabetic patients. *Nutrition Research*.

[22] Banihani S. A., Shuaibu S. M., Al-Husein B. A., Makahleh S. S. (2019). Fresh pomegranate juice decreases fasting serum erythropoietin in patients with type 2 diabetes. *International journal of food science*.

[23] Di Stefano V., Pitonzo R., Novara M. E. (2019). Antioxidant activity and phenolic composition in pomegranate (punica granatum l.) genotypes from south Italy by uhplc-orbitrap-ms approach. *Journal of the Science of Food and Agriculture*.

[24] Banihani S., Swedan S., Alguraan Z. (2013). Pomegranate and type 2 diabetes. *Nutrition Research*.

[25] Gil M. I., Tomás-Barberán F. A., Hess-Pierce B., Holcroft D. M., Kader A. A. (2000). Antioxidant activity of pomegranate juice and its relationship with phenolic composition and processing. *Journal of Agricultural and Food Chemistry*.

[26] Houston D. M. J., Bugert J. J., Denyer S. P., Heard C. M. (2017). Potentiated virucidal activity of pomegranate rind extract (pre) and punicalagin against herpes simplex virus (hsv) when co-administered with zinc (ii) ions, and antiviral activity of pre against hsv and aciclovir-resistant hsv. *PLoS One*.

[27] Su X., Sangster M. Y., D’Souza D. H. (2011). Time-dependent effects of pomegranate juice and pomegranate polyphenols on foodborne viral reduction. *Foodborne Pathogens and Disease*.

[28] Khalifa I., Nawaz A., Sobhy R., Althwab S. A., Barakat H. (2020). Polyacylated anthocyanins constructively network with catalytic dyad residues of 3CLpro of 2019-nCoV than monomeric anthocyanins: a structural-relationship activity study with 10 anthocyanins using in-silico approaches. *Journal of Molecular Graphics and Modelling*.

[29] Khalifa I., Zhu W., Mohammed H. H. H., Dutta K., Li C. (2020). Tannins inhibit sars-cov-2 through binding with catalytic dyad residues of 3cl(pro): an in silico approach with 19 structural different hydrolysable tannins. *Journal of Food Biochemistry*.

[30] Aviram M., Dornfeld L. (2001). Pomegranate juice consumption inhibits serum angiotensin converting enzyme activity and reduces systolic blood pressure. *Atherosclerosis*.

[31] Lynn A., Hamadeh H., Leung W. C., Russell J. M., Barker M. E. (2012). Effects of pomegranate juice supplementation on pulse wave velocity and blood pressure in healthy young and middle-aged men and women. *Plant Foods for Human Nutrition*.

[32] Shema-Didi L., Kristal B., Sela S., Geron R., Ore L. (2014). Does pomegranate intake attenuate cardiovascular risk factors in hemodialysis patients?. *Nutrition Journal*.

[33] Moazzen H., Alizadeh M. (2017). Effects of pomegranate juice on cardiovascular risk factors in patients with metabolic syndrome: a double-blinded, randomized crossover controlled trial. *Plant Foods for Human Nutrition*.

[34] Banihani S. A., Makahleh S. M., El-Akawi Z. J. (2020). Short-term effect of fresh pomegranate juice on serum cortisol and thyroxine in patients with type 2 diabetes. *Current Molecular Medicine*.

[35] Mourad J.-J., Levy B. I. (2020). Interaction between raas inhibitors and ace2 in the context of covid-19. *Nature Reviews Cardiology*.

[36] Hamming I., Timens W., Bulthuis M., Lely A., Navis G., van Goor H. (2004). Tissue distribution of ace2 protein, the functional receptor for sars coronavirus. A first step in understanding sars pathogenesis. *The Journal of Pathology*.

[37] Hoffmann M., Kleine-Weber H., Schroeder S. (2020). Sars-cov-2 cell entry depends on ace2 and tmprss2 and is blocked by a clinically proven protease inhibitor. *Cell*.

[38] Ferrario C. M., Jessup J., Chappell M. C. (2005). Effect of angiotensin-converting enzyme inhibition and angiotensin ii receptor blockers on cardiac angiotensin-converting enzyme 2. *Circulation*.

[39] Tipnis S. R., Hooper N. M., Hyde R., Karran E., Christie G., Turner A. J. (2000). A human homolog of angiotensin-converting enzyme. *Journal of Biological Chemistry*.

[40] Li Y., Zeng Z., Li Y. (2015). Angiotensin-converting enzyme inhibition attenuates lipopolysaccharide-induced lung injury by regulating the balance between angiotensin-converting enzyme and angiotensin-converting enzyme 2 and inhibiting mitogen-activated protein kinase activation. *Shock*.

[41] Wösten-van Asperen R. M., Lutter R., Specht P. A. (2011). Acute respiratory distress syndrome leads to reduced ratio of ace/ace2 activities and is prevented by angiotensin-(1-7) or an angiotensin ii receptor antagonist. *The Journal of Pathology*.

[42] Zheng Y.-Y., Ma Y.-T., Zhang J.-Y., Xie X. (2020). Reply to: ’Interaction between RAAS inhibitors and ACE2 in the context of COVID-19’. *Nature Reviews Cardiology*.

[43] Ding W., Li X., Wu W. (2018). [aliskiren inhibits angiotensin ii/angiotensin 1-7(ang ii/ang1-7) signal pathway in rats with diabetic nephropathy]. *Xi Bao Yu Fen Zi Mian Yi Xue Za Zhi*.

[44] Banihani S. A., Fashtaky R. A., Makahleh S. M., El‐Akawi Z. J., Khabour O. F., Saadeh N. A. (2019). Effect of fresh pomegranate juice on the level of melatonin, insulin, and fasting serum glucose in healthy individuals and people with impaired fasting glucose. *Food Sciences and Nutrition*.

[45] Das A. K., Mandal S. C., Banerjee S. K., Sinha S., Saha B. P., Pal M. (2001). Studies on the hypoglycaemic activity of punica granatum seed in streptozotocin induced diabetic rats. *Phytotherapy Research*.

[46] Hontecillas R., O’Shea M., Einerhand A., Diguardo M., Bassaganya-Riera J. (2009). Activation of PPAR *γ* and *α* by punicic acid ameliorates glucose tolerance and suppresses obesity-related inflammation. *Journal of the American College of Nutrition*.

[47] Johanningsmeier S. D., Harris G. K. (2011). Pomegranate as a functional food and nutraceutical source. *Annual Review of Food Science and Technology*.

[48] Fuhrman B., Volkova N., Aviram M. (2010). Pomegranate juice polyphenols increase recombinant paraoxonase-1 binding to high-density lipoprotein: studies in vitro and in diabetic patients. *Nutrition*.

[49] Benalla W., Bellahcen S., Bnouham M. (2010). Antidiabetic medicinal plants as a source of alpha glucosidase inhibitors. *Current Diabetes Reviews*.

[50] Bektas N., Ozturk Y. (2012). Effect of phenolic acids on functions of rat aorta, vas deferens and on metabolic changes in streptozotocin-induced diabetes. *Indian Journal of Pharmacology*.

[51] Alzoubi K. H., Hasan Z. A., Khabour O. F. (2018). The effect of high-fat diet on seizure threshold in rats: role of oxidative stress. *Physiology & Behavior*.

[52] Banihani S. A. (2018). Effect of coenzyme q10 supplementation on testosterone. *Biomolecules*.

[53] Mohan M., Waghulde H., Kasture S. (2010). Effect of pomegranate juice on angiotensin ii-induced hypertension in diabetic wistar rats. *Phytotherapy Research*.

[54] Makino-Wakagi Y., Yoshimura Y., Uzawa Y., Zaima N., Moriyama T., Kawamura Y. (2012). Ellagic acid in pomegranate suppresses resistin secretion by a novel regulatory mechanism involving the degradation of intracellular resistin protein in adipocytes. *Biochemical and Biophysical Research Communications*.

[55] Degawa-Yamauchi M., Bovenkerk J. E., Juliar B. E. (2003). Serum resistin (fizz3) protein is increased in obese humans. *Journal of Clinical Endocrinology & Metabolism*.

[56] Rozenberg O., Howell A., Aviram M. (2006). Pomegranate juice sugar fraction reduces macrophage oxidative state, whereas white grape juice sugar fraction increases it. *Atherosclerosis*.

[57] Schubert S. Y., Neeman I., Resnick N. (2002). A novel mechanism for the inhibition of NF*κ*B activation in vascular endothelial cells by natural antioxidants. *The FASEB Journal*.

[58] Aggarwal B. B., Shishodia S. (2004). Suppression of the nuclear factor-*κ*b activation pathway by spice-derived phytochemicals: reasoning for seasoning. *Annals of the New York Academy of Sciences*.

[59] Basu A., Penugonda K. (2009). Pomegranate juice: a heart-healthy fruit juice. *Nutrition Reviews*.

[60] Asgary S., Keshvari M., Sahebkar A., Sarrafzadegan N. (2017). Pomegranate consumption and blood pressure: a review. *Current Pharmaceutical Design*.

[61] Barati Boldaji R., Akhlaghi M., Sagheb M. M., Esmaeilinezhad Z. (2020). Pomegranate juice improves cardiometabolic risk factors, biomarkers of oxidative stress and inflammation in hemodialysis patients: a randomized crossover trial. *Journal of the Science of Food and Agriculture*.

[62] Mazani M., Fard A. S., Baghi A. N., Nemati A., Mogadam R. A. (2014). Effect of pomegranate juice supplementation on matrix metalloproteinases 2 and 9 following exhaustive exercise in young healthy males. *JPMA. The Journal of the Pakistan Medical Association*.

[63] Aksu D. S., Sağlam Y. S., Yildirim S., Aksu T. (2017). Effect of pomegranate (punica granatum l.) juice on kidney, liver, heart and testis histopathological changes, and the tissues lipid peroxidation and antioxidant status in lead acetate-treated rats. *Cellular and Molecular Biology*.

[64] Estrada-Luna D., Martínez-Hinojosa E., Cancino-Diaz J. C., Belefant-Miller H., López-Rodríguez G., Betanzos-Cabrera G. (2018). Daily supplementation with fresh pomegranate juice increases paraoxonase 1 expression and activity in mice fed a high-fat diet. *European Journal of Nutrition*.

[65] Kojadinovic M. I., Arsic A. C., Debeljak-Martacic J. D. (2017). Consumption of pomegranate juice decreases blood lipid peroxidation and levels of arachidonic acid in women with metabolic syndrome. *Journal of the Science of Food and Agriculture*.

[66] Taheri Rouhi S. Z., Sarker M. M. R., Rahmat A., Alkahtani S. A., Othman F. (2017). The effect of pomegranate fresh juice versus pomegranate seed powder on metabolic indices, lipid profile, inflammatory biomarkers, and the histopathology of pancreatic islets of langerhans in streptozotocin-nicotinamide induced type 2 diabetic sprague-dawley rats. *BMC Complementary and Alternative Medicine*.

[67] Les F., Prieto J. M., Arbonés-Mainar J. M., Valero M. S., López V. (2015). Bioactive properties of commercialised pomegranate (punica granatum) juice: antioxidant, antiproliferative and enzyme inhibiting activities. *Food & Function*.

[68] Rosenblat M., Volkova N., Coleman R., Aviram M. (2006). Pomegranate byproduct administration to apolipoprotein e-deficient mice attenuates atherosclerosis development as a result of decreased macrophage oxidative stress and reduced cellular uptake of oxidized low-density lipoprotein. *Journal of Agricultural and Food Chemistry*.

[69] Rosenblat M., Volkova N., Abassi Z., Britton S. L., Koch L. G., Aviram M. (2015). High intrinsic aerobic capacity and pomegranate juice are protective against macrophage atherogenecity: studies in high- vs. Low-capacity runner (hcr vs. Lcr) rats. *The Journal of Nutritional Biochemistry*.

[70] Aviram M., Rosenblat M., Gaitini D. (2004). Pomegranate juice consumption for 3 years by patients with carotid artery stenosis reduces common carotid intima-media thickness, blood pressure and ldl oxidation. *Clinical Nutrition*.

[71] Mortada W. I., Awadalla A., Khater S. M., Barakat N. M., Husseiny S. M., Shokeir A. A. (2020). Preventive effect of pomegranate juice against chemically induced bladder cancer: an experimental study. *Heliyon*.

[72] Paller C. J., Pantuck A., Carducci M. A. (2017). A review of pomegranate in prostate cancer. *Prostate Cancer and Prostatic Diseases*.

[73] Sharma P., McClees S. F., Afaq F. (2017). Pomegranate for prevention and treatment of cancer: an update. *Molecules*.

[74] Malik A., Afaq F., Sarfaraz S., Adhami V. M., Syed D. N., Mukhtar H. (2005). Pomegranate fruit juice for chemoprevention and chemotherapy of prostate cancer. *Proceedings of the National Academy of Sciences*.

[75] Naiki-Ito A., Chewonarin T., Tang M. (2015). Ellagic acid, a component of pomegranate fruit juice, suppresses androgen-dependent prostate carcinogenesis via induction of apoptosis. *The Prostate*.

[76] Afaq F., Zaid M. A., Khan N., Dreher M., Mukhtar H. (2009). Protective effect of pomegranate-derived products on uvb-mediated damage in human reconstituted skin. *Experimental Dermatology*.

[77] Adhami V. M., Khan N., Mukhtar H. (2009). Cancer chemoprevention by pomegranate: Laboratory and clinical evidence. *Nutrition and Cancer*.

[78] Jaganathan S. K., Vellayappan M. V., Narasimhan G., Supriyanto E. (2014). Role of pomegranate and citrus fruit juices in colon cancer prevention. *World Journal of Gastroenterology*.

[79] Saruwatari A., Okamura S., Nakajima Y., Narukawa Y., Takeda T., Tamura H. (2008). Pomegranate juice inhibits sulfoconjugation in caco-2 human colon carcinoma cells. *Journal of Medicinal Food*.

[80] Adams L. S., Seeram N. P., Aggarwal B. B., Takada Y., Sand D., Heber D. (2006). Pomegranate juice, total pomegranate ellagitannins, and punicalagin suppress inflammatory cell signaling in colon cancer cells. *Journal of Agricultural and Food Chemistry*.

[81] Sturgeon S. R., Ronnenberg A. G. (2010). Pomegranate and breast cancer: possible mechanisms of prevention. *Nutrition Reviews*.

[82] Rocha A., Wang L., Penichet M., Martins-Green M. (2012). Pomegranate juice and specific components inhibit cell and molecular processes critical for metastasis of breast cancer. *Breast Cancer Research and Treatment*.

[83] Mehta R., Lansky E. P. (2004). Breast cancer chemopreventive properties of pomegranate (punica granatum) fruit extracts in a mouse mammary organ culture. *European Journal of Cancer Prevention*.

[84] Khan G. N., Gorin M. A., Rosenthal D. (2009). Pomegranate fruit extract impairs invasion and motility in human breast cancer. *Integrative Cancer Therapies*.

[85] Seeram N., Adams L., Henning S. (2005). In vitro antiproliferative, apoptotic and antioxidant activities of punicalagin, ellagic acid and a total pomegranate tannin extract are enhanced in combination with other polyphenols as found in pomegranate juice. *The Journal of Nutritional Biochemistry*.

[86] Derakhshan Z., Ferrante M., Tadi M. (2018). Antioxidant activity and total phenolic content of ethanolic extract of pomegranate peels, juice and seeds. *Food and Chemical Toxicology*.

[87] Wang L., Ho J., Glackin C., Martins-Green M. (2012). Specific pomegranate juice components as potential inhibitors of prostate cancer metastasis. *Translational Oncology*.

[88] Banerjee N., Kim H., Talcott S., Mertens-Talcott S. (2013). Pomegranate polyphenolics suppressed azoxymethane-induced colorectal aberrant crypt foci and inflammation: possible role of mir-126/vcam-1 and mir-126/pi3k/akt/mtor. *Carcinogenesis*.

[89] Koyama S., Cobb L. J., Mehta H. H. (2010). Pomegranate extract induces apoptosis in human prostate cancer cells by modulation of the igf-igfbp axis. *Growth Hormone & IGF Research*.

[90] Sartippour M., Seeram N., Rao J. (2008). Ellagitannin-rich pomegranate extract inhibits angiogenesis in prostate cancer in vitro and in vivo. *International Journal of Oncology*.

[91] Seeram N. P., Aronson W. J., Zhang Y. (2007). Pomegranate ellagitannin-derived metabolites inhibit prostate cancer growth and localize to the mouse prostate gland. *Journal of Agricultural and Food Chemistry*.

[92] Dahlawi H., Jordan‐Mahy N., Clench M., McDougall G. J., Maitre C. L. (2013). Polyphenols are responsible for the proapoptotic properties of pomegranate juice on leukemia cell lines. *Food Sciences and Nutrition*.

[93] Wu S., Tian L. (2017). Diverse phytochemicals and bioactivities in the ancient fruit and modern functional food pomegranate (punica granatum). *Molecules*.

[94] Liu H., Zeng Z., Wang S. (2017). Main components of pomegranate, ellagic acid and luteolin, inhibit metastasis of ovarian cancer by down-regulating mmp2 and mmp9. *Cancer Biology & Therapy*.

[95] Wang L., Li W., Lin M. (2014). Luteolin, ellagic acid and punicic acid are natural products that inhibit prostate cancer metastasis. *Carcinogenesis*.

